# A new method of intraoperative pelvic neuromonitoring: a preclinical feasibility study in a porcine model

**DOI:** 10.1038/s41598-022-07576-8

**Published:** 2022-03-07

**Authors:** Ramona Schuler, Matthias Goos, Andreas Langer, Maximilian Meisinger, Christoph Marquardt, Helga Fritsch, Marko Konschake

**Affiliations:** 1 Research and Development, Dr. Langer Medical GmbH, Waldkirch, Germany; 2 Institute of Biomedical Engineering and Informatics, TU Ilmenau, Ilmenau, Germany; 3 Department of General and Visceral Surgery, Medical Center - University of Freiburg and the Center for Experimental Models and Transgenic Service, Freiburg im Breisgau, Germany; 4 Department of General, Visceral, Thoracic and Pediatric Surgery, Ludwigsburg Hospital, Ludwigsburg, Germany; 5 Department of Anatomy, Histology and Embryology, Institute of Clinical and Functional Anatomy, Medical University of Innsbruck (MUI), Müllerstr. 59, 6020 Innsbruck, Austria

**Keywords:** Bladder, Translational research, Bladder, Autonomic nervous system

## Abstract

Low anterior resections (LAR) are frequently associated with complications such as urinary and fecal incontinence as well as sexual disorders. Typical risk factors are rectal cancer with low tumor location, preoperative radiotherapy, and surgery-related damage of pelvic autonomic nerves. As preserving the pelvic autonomic nerves without any technical assistance is challenging, the objective of this preclinical study was to investigate the technical feasibility of a new method for intraoperative pelvic neuromonitoring. Twelve female pigs undergoing low anterior resections were involved in a prospective preclinical study. Intraoperative pelvic neuromonitoring included direct pelvic nerve stimulation and tissue impedance measurement on the urinary bladder and the rectum for the identification of efferent pelvic nerves in the surgical area. Immunohistochemistry was used to verify the results. Smooth muscle contraction of the urinary bladder and/or the rectum in response to direct stimulation of the innervating nerves was detectable with impedance measurement. The macroscopic contraction of both the urinary bladder and the rectum correlated with a change in tissue impedance compared to the status before contraction. Thus, it was possible to identify pelvic nerves in the surgical area, which allows the nerves to be preserved. The results indicate a reliable identification of pelvic autonomic nerves, which allows nerve damage to be prevented in the future.

## Introduction

Knowledge of pelvic neuroanatomy is crucial for rectal surgery as pelvic nerves are a complex system of autonomic, sensory and motor nerves^1–5^. Pelvic nerves ensure the innervated organs’ function, including the urinary bladder, the rectum and the sexual organs. Hence, preserving the superior and inferior hypogastric nerves and plexuses from damage during surgery in the lesser pelvis is not only essential but also required by national guidelines for the treatment of colorectal cancer^6^. Especially after low anterior resection (LAR) with total mesorectal excision (TME) for rectal cancer, damage to the above-mentioned autonomic nerves may lead to urinary and fecal incontinence as well as sexual disorders, which lowers the quality of life (QOL) of the patients dramatically. The complication known as Low Anterior Resection Syndrome (LARS) describes fecal continence disorders and is measured by the validated LARS score, a numerical value from 0 to 42 points, where a score above 30 points is regarded as „major LARS “, signifying major fecal continence problems. Risk factors for LARS are rectal cancer, especially with low location of the tumor, surgery-related neural damage and preoperative radiotherapy (RTx)^7^. Recent studies report on symptoms of intestinal dysfunctions such as incontinence, diarrhea and increased frequencies of defecation in 30–80% of the patients^7–11^. The QOL reduction is detrimental to the patients’ well-being and therefore leads to high costs for the healthcare system.

Preventing the pelvic plexus from damage without any technical assistance is challenging for the surgeon during LAR with TME in adverse anatomical situations (e.g. male pelvis, low rectal cancer, high BMI). The reason for this is that it is difficult to identify the pelvic nerves and differentiate them from other tissue due to their delicate and fragile phenotype and interindividual differences in their anatomical position. Pelvic nerves can be macroscopically similar to connective tissue or fat tissue and thus hardly visible. The complexity of the plexus and the mixture of sympathetic and parasympathetic pathways in the autonomic system are further issues^1,5,12,13^. Hence, the standard methods of intraoperative neuromonitoring which are known e.g. from spinal and neurosurgery are hardly applicable^14,15^.

Standard methods of intraoperative neuromonitoring primarily include electromyography (EMG) and evoked potentials (EP), such as somatosensory evoked potentials (SSEP), auditory evoked potentials (AEP), visual evoked potentials (VEP), and motor evoked potentials (MEP)^16,17^. Cortical evoked potentials are stimulated and recorded for monitoring and functional control of afferent sensory nerves (e.g., median and tibial nerves, vestibulocochlear nerve, optic nerve, and visual pathway). The adaptation to pelvic autonomic nerves includes stimulation and recording of pudendal nerve SSEPs, but identification of pelvic autonomic nerves as the hypogastric plexus in the surgical area is not possible^18^. Motor evoked potentials (MEPs) are generated by transcranial stimulation of the motor cortex. The method allows monitoring of the integrity of efferent motor pathways. When applied to the pelvic nerves, the MEP method can be used to monitor innervation of the external urethral sphincter (EUS) and external anal sphincter (EAS), both of which are motor muscles innervated by the pudendal nerve^16,17^. The pudendal nerve is not at risk during low anterior resections because it is located deep in the posterior region.

Electromyography (EMG) is based on the principle that each stimulation pulse applied to the tissue with a handheld probe produces a compound muscle action potential (CMAP) in the innervated motor muscle if the nerve is intact. The CMAPs are recorded by electromyography (EMG) and interpreted by the surgeon. Motor muscles twitch with a latency of a few milliseconds (depending on the distance between stimulation and recording position) after a stimulation pulse, and the refractory period is again a few milliseconds after a subsequent CMAP can be measured^16,17^. In contrast, smooth muscles contract in slow waves after stimulation of autonomic nerves for several seconds and require a similar relaxation phase. Due to the different contraction and excitation behavior of motor and smooth muscles, a different measurement technique consisting of signal amplification and signal processing is required, the EMG method for motor muscles is not suitable for smooth muscles. The research group Kauff and Kneist et al. investigated and developed a method for intraoperative neuromonitoring of pelvic autonomic nerves based on adapted smooth muscle EMG measurement in combination with bladder manometry^19–21^. This is the only method on the market that has the same intended purpose as the method presented in this study. The method is based on the principle of direct stimulation of the pelvic nerves with a handheld probe in the surgical field and EMG measurement on the internal anal sphincter (IAS) and bladder manometry. Because EMG measurement (as applied to motor function) is not applicable to the autonomic system, an adaptation of signal amplification and signal processing was made. Increased EMG activity was measured at the IAS during the period of direct nerve stimulation, extracted from the signal using frequency analysis^15^. EMG measurement is performed on the IAS, but not on the urinary bladder. Bladder response is measured with manometry, which requires bladder filling with Ringer's solution for each iterative stimulation period, otherwise no bladder contraction can be detected. The impedance measurement method presented in this study does not require bladder filling and constitutes a direct and easily interpretable indicator of the activity of the empty urinary bladder and the rectum. This study aimed to develop a new approach to intraoperative pelvic neuromonitoring for the reliable identification of pelvic nerves to ensure nerve preservation and a better outcome after LAR with TME. The objective of this study was to investigate the technical feasibility of a new method for intraoperative pelvic neuromonitoring.

## Methods

The study was a prospective preclinical animal study, which included twelve female pigs. The study was approved by the Regional Administrative Council in Freiburg (G-16-127) and was conducted at the Center of Experimental Models and Transgenic Service (CEMT) in Freiburg. All methods were performed in accordance with the relevant guidelines and regulations, the study was conducted in compliance with the ARRIVE guidelines and the directive 2010/63/EU. The pigs (mean weight 70 kg) underwent LAR in an open surgical procedure. Total intravenous anesthesia (TIVA) was performed with propofol for initiation and fentanyl for anesthesia maintenance. In four cases the influence of muscle relaxation on intraoperative neuromonitoring was tested with intravenous vecuronium. After conducting and evaluating pelvic neuromonitoring, the animals were euthanized under deep anesthesia using intravenous potassium chloride (KCl).

### Neuromonitoring principle

Intraoperative neuromonitoring is based on the electrophysiological principle of direct stimulation of nerves in the surgical field and electrophysiological recording of the response of the target organs. An electric current is applied to the tissue with a handheld probe, which generates an electric field in the tissue depending on the strength of the applied stimulation current. The nerves reached by the electric field generate action potentials when their excitation threshold is exceeded. The action potentials of the nerves are transmitted to the target muscle, resulting in muscle contraction.

In the motor system, each stimulation pulse generates a compound muscle action potential (CMAP), which is measured by electromyography (EMG). The surgeon interprets the stimulus synchronized CMAPs, which provide information about the anatomical location and integrity of the stimulated nerve(s). In the autonomic system, nerve excitation does not result in stimulus synchronous CMAPs, but in modulation of smooth muscle activity, which results in smooth muscle contraction in slow waves. The slow smooth muscle contraction occurs after the application of stimulation pulses to the nerve tissue for several seconds. Slow wave contraction is measured by a change in tissue impedance compared to the pre-contraction state.

### Used neuromonitoring technology

**Figure 1:**
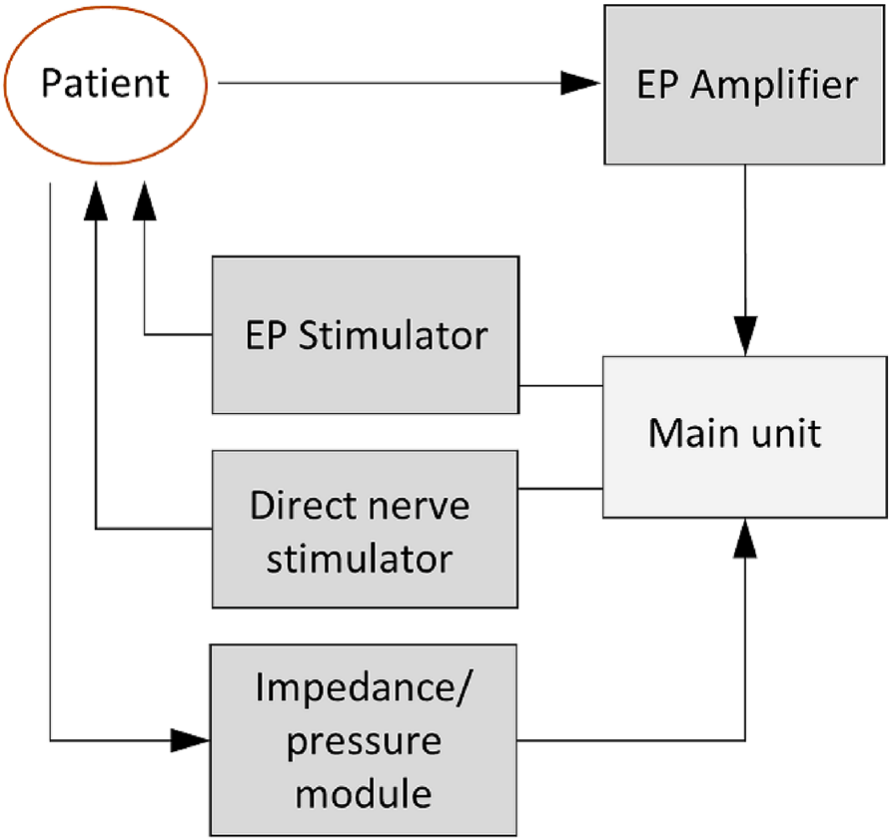
Components of the prototype of the neuromonitoring system AVALANCHE NeuroNeB for intraoperative pelvic neuromonitoring in a schematic. Direct nerve stimulator, impedance and pressure module are used for intraoperative identification of efferent pelvic autonomic nerves. Additional components are the EP stimulator and EP amplifier. The main unit controls the components.

The prototype of a new neuromonitoring system AVALANCHE NeuroNeB (Dr. Langer Medical GmbH, Waldkirch, Germany, see Fig. 1) consists of the following components: a main unit (PC), a constant current stimulator for direct nerve stimulation, a two-channel impedance measurement module for displaying voltage drop across tissue (proportional to tissue impedance) as a function of time, a control for a pressure transducer for monitoring physiological pressure changes, a constant current stimulator for stimulating evoked potentials (EP stimulator) and an amplifier module for recording evoked potentials (EP amplifier). The measurement of viscerally evoked potentials for the verification of the functional integrity of the afferent pelvic nerves is not part of this study.

**Figure 2:**
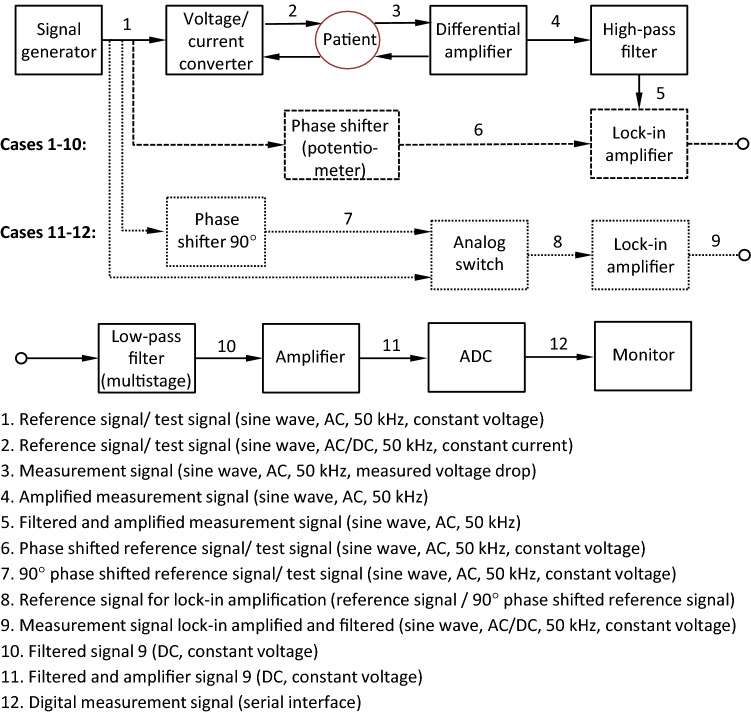
Block diagram of the impedance measurement module of the AVALANCHE NeuroNeB prototype. A constant current sine wave is applied to the tissue. Voltage drop across the tissue is measured and high pass filtered. Lock-in amplification with a manually phase shifted reference signal (case 1–10—dashed line) or alternated with a sine and a cosine reference signal (case 11 and 12—dotted line) followed. The low pass filtered and amplified signal is fed into an analog-digital-converter and displayed on the neuromonitor, representing tissue impedance.

The impedance measurement module employed to display changes in tissue impedance is a new technology in neuromonitoring^17^ and therefore explained in more detail in the following, see Fig. 2. The module consists of a current source, which is supplied with a sine reference voltage from a signal generator. The delivered alternating constant current (test current) of 50 µA is applied to the tissue. The voltage drop across the tissue is measured with a differential amplifier.

The impedance measurement module was developed in two versions. Version one, which was used in cases one to ten in the porcine model, requires the usage of two additional electrodes to measure the voltage drop (necessitating four electrodes for each organ). In version two, which was employed in cases eleven and twelve, the number of electrodes is reduced to two for each organ to reduce invasiveness and to improve the applicability of the method. In this setup the electrodes are used for both applying the test current and measuring the voltage drop.

The measured voltage drop across the tissue, in the following referred to as “measurement signal”, is amplified (gain of 50), high-pass filtered (cutoff frequency of 160 Hz) and delivered to a lock-in amplifier. The reference voltage from the signal generator is used as reference signal for the lock-in amplification.

Because of the capacitive characteristics of living tissue there is a phase shift between the measurement signal and the reference signal^22^. For phase adjustment in version one the reference signal is shifted in phase manually so that measurement signal and reference signal are in phase. Therefore, the measurement signal and the test signal were measured intraoperatively using alligator clips on the electrodes and an oscilloscope. Using a potentiometer in a phase shift circuit between the test signal generator and the lock-in amplifier, the phase shift between the two signals was corrected by adjusting the potentiometer. This is followed by lock-in amplification. Demodulation (signal integration) is realized via a 3rd-order low-pass filter (lowest cutoff frequency of 5 Hz), resulting in a direct current (DC) signal. The DC signal is amplified (gain of 11) and fed into an analog–digital converter (ADC). The output signal from the ADC is displayed on the neuromonitor as a function of time, representing tissue impedance.

To improve the applicability of the method in a clinical setting, phase adjustment in version two is achieved via an alternating input of the sine reference signal and a 90°-phase-shifted second reference signal (a cosine reference signal) to the lock-in amplifier. This is followed by alternating multiplication of the measurement signal with the sine and the cosine reference signal, resulting in two output signal components. Both signals are demodulated and amplified as in version one, which is described above.

The output signals of the ADC are vectorially added to a resultant, which allows the actual phase shift of the measurement signal to be calculated due to capacitive tissue characteristics. The resultant is displayed on the neuromonitor as a function of time, representing tissue impedance.

### Application of the intraoperative neuromonitoring setup

Direct nerve stimulation was performed with a bipolar handheld stimulation probe (REF 40-0016-SP, Spes Medica S.r.l, Italy) connected to the direct nerve stimulator. Additionally, a stimulation electrode (Saxophone electrode, Dr. Langer Medical GmbH, Waldkirch, Germany) was placed permanently at a fixed position to ensure steady test conditions. Stimulation is performed with a constant current stimulator (maximum 100 mA/150 V). Monophasic rectangular pulses in the range of 10–50 mA with 1000 µs pulse width and a pulse frequency of 10–50 Hz were applied. The influence of the stimulation current and the stimulation frequency on the amplitude of the tissue impedance change was evaluated.

Three different stimulation positions were defined between the target organs and the sacral nerve roots: First, direct stimulation of the detrusor muscle or rectum muscle was conducted to verify the experimental setup. Second, the stimulation of the vesicouterine ligament (urinary bladder) and paraproctium (rectum) with the Saxophone electrode was carried out to have a fixed landmark close to the target organ, which ensures comparable test conditions between individuals. Third, the superior and inferior hypogastric plexuses were stimulated with the handheld stimulation probe to identify the plexus and its branches, as it is required in a clinical setting.

For impedance measurement on the target organs a bladder catheter including two urethral surface electrodes (Disposable Urethral Catheter Electrode, Spes Medica S.r.l, Italy), monopolar needle electrodes (SEI EMG S.r.l, Italy) and a bipolar rectal probe with surface electrodes (PE0001, Shenzhen Med-link Electronics Tech Co., Ltd., China) were used. As explained above, surgery in cases one to ten was performed using four electrodes on each target organ, while in cases eleven and twelve only two electrodes each were used.

**Figure 3:**
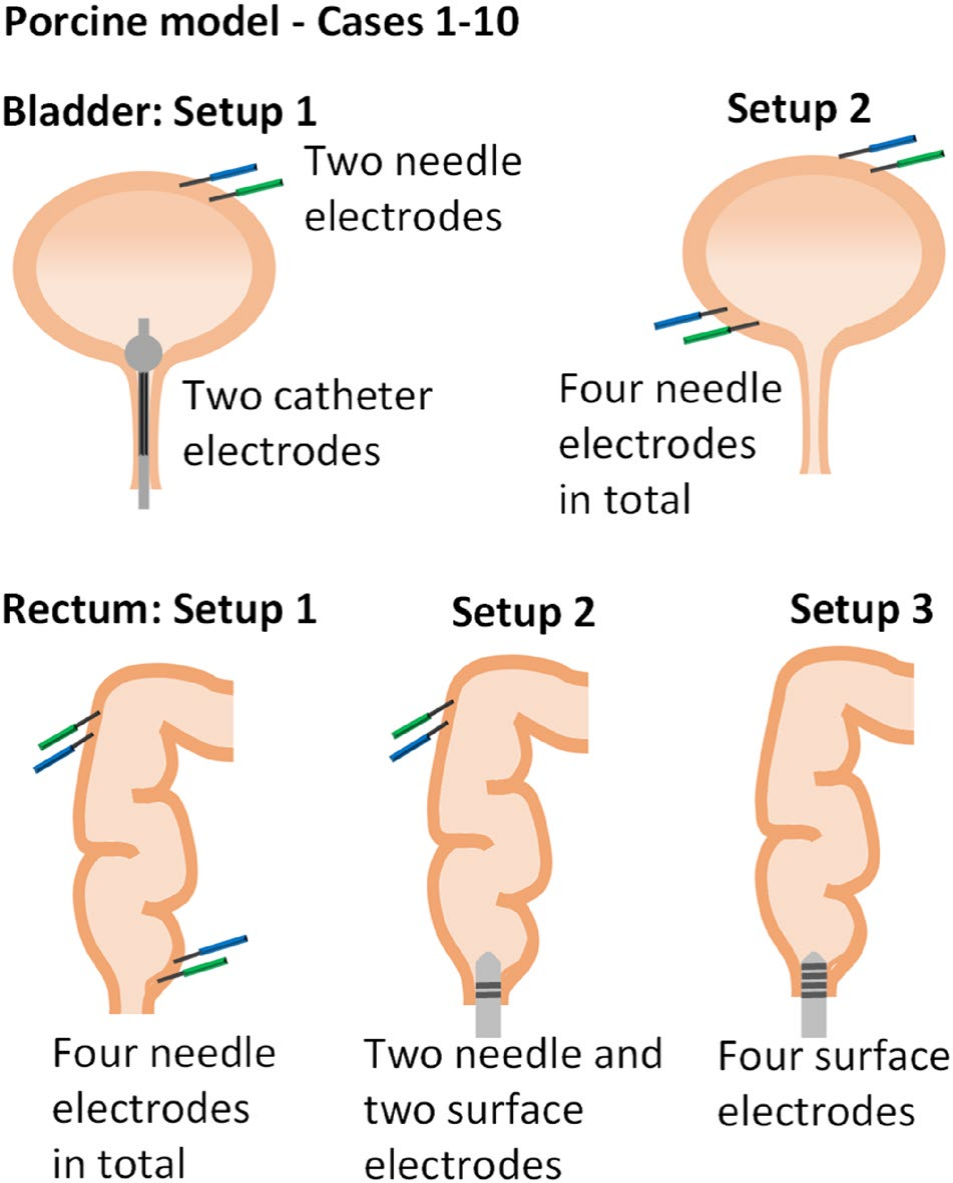
Schematic of the electrode positioning setups for impedance measurement on the urinary bladder and rectum. In cases one to ten four electrodes per organ were needed, in cases eleven and twelve two electrodes per organ were used. Urinary bladder: Setup 1 uses needle electrodes and catheter electrodes; Setup 2 uses needle electrodes only. Rectum: Setup 1 uses needle electrodes only, Setup 2 uses needle electrodes and surface electrodes on a rectal probe, Setup 3 uses surface electrodes only.

Different electrode setups consisting of either needle electrodes only, or a combination of needle electrodes and surface electrodes were evaluated, see the schematic in Fig. 3. Thereby, one needle electrode or electrode pair was positioned on the proximal portion of the organ (urinary bladder’s apex, upper rectum), and the other needle or surface electrode or electrode pair was used on the distal portion of the organ (urinary bladder’s vertex, lower rectum). The needle electrodes were fixed with a few stitches. The surface electrodes (rectal probe and urethral catheter) are not fixed and remain in position because of their dedicated design.

In case 1–10 (impedance measurement with four electrodes) one electrode at the proximal part of the organ and one electrode at the distal part of the organ were used to apply the test signal. The outer electrodes are best for applying the test signal. In addition, one electrode at the proximal part of the organ and one electrode at the distal part of the organ were used to record the test signal. The inner electrodes are best suited as measurement electrodes. In cases 11–12, impedance measurement was performed with two electrodes in total, both the application of the test signal and the recording of the measurement signal are performed with the same electrodes.

As a reference method for the impedance measurement on the detrusor muscle intraoperative manometry of the urinary bladder was performed, which required the bladder to be filled with 150 ml of Ringer’s solution. For this purpose either a suprapubic or a transurethral catheter as well as a pressure transducer and Luer-Lock connections to the bladder catheter (Monitoring Set, HJK Sensoren + Systeme GmbH & Co. KG, Germany) were used.

### Signal analysis

The results of the study were analyzed and comparisons between the animals were made according to the electrode setup, the stimulation site and the stimulation parameters. The arithmetic mean and median values were calculated, and the dispersion of the values was analyzed in a box plot to determine the optimal parameters and setups. While high arithmetic mean and median values were considered positive, a high dispersion of values was considered negative. Furthermore, practical aspects of the application to humans in a clinical setting were taken into account. Additionally, the macroscopic contraction of the smooth muscle was examined visually to determine the correlation with the results of the impedance measurement. To exclude artefacts, a negative control was performed after deliberately cutting nerves.

All tests were performed without the administration of a muscle relaxant in all cases. In four of the twelve cases, the tests were repeated to analyze the influence of the muscle relaxant (vecuronium) on the results.

### Immunohistochemistry

Immunohistochemistry was used for nerve tissue verification of the results of the impedance measurement using S100, VIP, DBH and TH antibodies. Tissue samples which were identified as nerve tissue by means of impedance measurement and direct nerve stimulation were obtained for immunohistochemistry. The samples were excised and then promptly fixed in 4% paraformaldehyde (PFA) in phosphate buffered saline (PBS, 0.1 M) at a pH of 7.4 and left overnight. Subsequently, the samples were rinsed in PBS and were then prepared by dehydrating and later embedding in paraffin using a histological infiltration processor (Miles Scientific Inc., Naperville, IL, USA). Sequential sections of 4 μm thickness were made on a HM 355S microtome (Microm, Walldorf, Germany) and affixed on SuperFrost Plus slides (Menzel, Braunschweig, Germany). The affixed specimens were dried overnight at room temperature. Afterwards, the section-containing slides were incubated at 60 °C for 2 h to adhere the sectioned specimens firmly onto them.

Immunohistochemistry was rendered with a Ventana Roche Discovery XT Immunostainer (Mannheim, Germany), using a DAB-MAP discovery research standard procedure. The mounted sections were incubated with the appropriate primary antibody at 37 °C for 1 h. Following this, the specimens were incubated with Discovery Universal Secondary Antibody, Ventana 760–4250 at room temperature for 30 min. Antibody detection was attained with the DAB-MAP Detection Kit (Ventana 760–124) using a combinatorial approach involving the diaminobenzidine development method with copper enhancement followed by light counter staining with haematoxylin (Ventana 760–2021) for 4 min. The stained sections were then manually dehydrated using an upgraded alcohol series, clarified with xylene and then mounted permanently with Entellan (Merck, Darmstadt, Germany).

The entire immunohistochemical staining reaction was benchmarked against appositive controls (e.g. small intestine, brain, and pancreas). Auxiliary negative controls were acquired by alternating the primary antibodies with reaction buffer or substituting them with isotype matching immunoglobulins.

### Cadaver models

In order to evaluate the feasibility of the method on humans, the pelvic plexus was macroscopically dissected on human cadavers and the potential electrode positions for a clinical setting were evaluated.

The body-donor to science was donated to the anatomical department of Anatomy, Histology and Embryology, Institute of Clinical and Functional Anatomy, Medical University of Innsbruck (MUI). The individuals had given their written informed consent before death for use for scientific and educational purposes. Under national law, scientific institutions (generally medical university institutes, departments, or divisions) are entitled to receive the body after death primarily through a specific legacy, which is a special form of last will and testament. Legacies are not accepted without the donor having recorded his legacy and given the appropriate information on which to make a decision based on written informed consent (ethics policy). Therefore, approval by the ethics committee was not necessary.

### Ethics declarations

We confirm that the study was fully approved by the ethics commitee of the Regional Administrative Council in Freiburg (G-16-127) and was conducted at the Center of Experimental Models and Transgenic Service (CEMT) in Freiburg. We confirm that all methods were carried out in accordance with relevant guidelines and regulations. According to the ethical statements we want to clarify that an ethics review board approval for the body-donors used is not necessary (and available) by Austrian laws (according to Tyrolean Sanitary Law §§28–32). The body-donor to science was donated to the anatomical department of Anatomy, Histology and Embryology, Institute of Clinical and Functional Anatomy, Medical University of Innsbruck (MUI). The individuals had given their written informed consent before death for use for scientific and educational purposes. Under national law, scientific institutions (generally medical university institutes, departments, or divisions) are entitled to receive the body after death primarily through a specific legacy, which is a special form of last will and testament. Legacies are not accepted without the donor having recorded his legacy and given the appropriate information on which to make a decision based on written informed consent (ethics policy). Therefore, approval by the ethics committee was waived. All processes in getting these body-donors to science (after obtaining informed consent) are either described in the manuscript or in detail in the references below^31,32^: Homepages (only available in German language): https://www.anatomie-innsbruck.at/koerperspende/information-zu-ihrer-koerperspende/. https://www.ris.bka.gv.at/GeltendeFassung.wxe?Abfrage=LrT&Gesetzesnummer=20000193.

## Results

Direct nerve stimulation for the excitation of the pelvic autonomic nerves led to a contraction of the innervated organs, which was recorded as a change in tissue impedance of the smooth muscles. Thus, it became possible to identify the pelvic nerves which innervate the urinary bladder and the rectum, making their preservation feasible. Direct nerve stimulation and impedance measurement on the urinary bladder were conducted in eleven cases, on the rectum in ten cases. In all cases contractions of the smooth muscle resulted in a change in tissue impedance, which allows nerve identification and preservation.

While the change in tissue impedance on the target organ is an indicator for smooth muscle contraction and thereby for the functional integrity of the nerves, the absolute tissue impedance value is not relevant. Therefore, a qualitative evaluation of the tissue impedance during muscle contraction compared to the status before contraction was applied. For this purpose, the output signal of the above-described AVALANCHE NeuroNeB prototype was normalized to the level of the status before contraction. Thus, the qualitative evaluation resulted in a dimensionless output signal U(t)/U(0), whereby U(t) is proportional to the tissue impedance during muscle contraction and U(0) is proportional to the tissue impedance before muscle contraction.

There is a difference between the function of a smooth muscle (visceromotor response) and that of a skeletal muscle (somatomotor response). While a skeletal muscle responds to stimulation of the innervated nerves with twitching, the smooth muscle responded to the ongoing nerve stimulation of autonomic nerves with a steady and slow contraction to its maximum. After maximal contraction, the complete relaxation of the smooth muscle required a pause phase. The degree of relaxation was determined visually, depending on the macroscopic contraction of the muscle. In terms of the visual observation of smooth muscle contraction the porcine model is ideal because the porcine bladder is less covered and grown together with surrounding tissue than the human urinary bladder. A pause phase of about two minutes was found to be adequate to achieve complete relaxation—a state in which no further macroscopic contraction was observed. When subsequent stimulation started before complete relaxation, the maximum contraction seen before could not be reached. Therefore, a stimulation protocol with defined stimulation and relaxation phases was used to guarantee valid and comparable results. It consisted of three phases including 30-s continuous stimulation and two-minute relaxation without stimulation.

**Figure 4:**
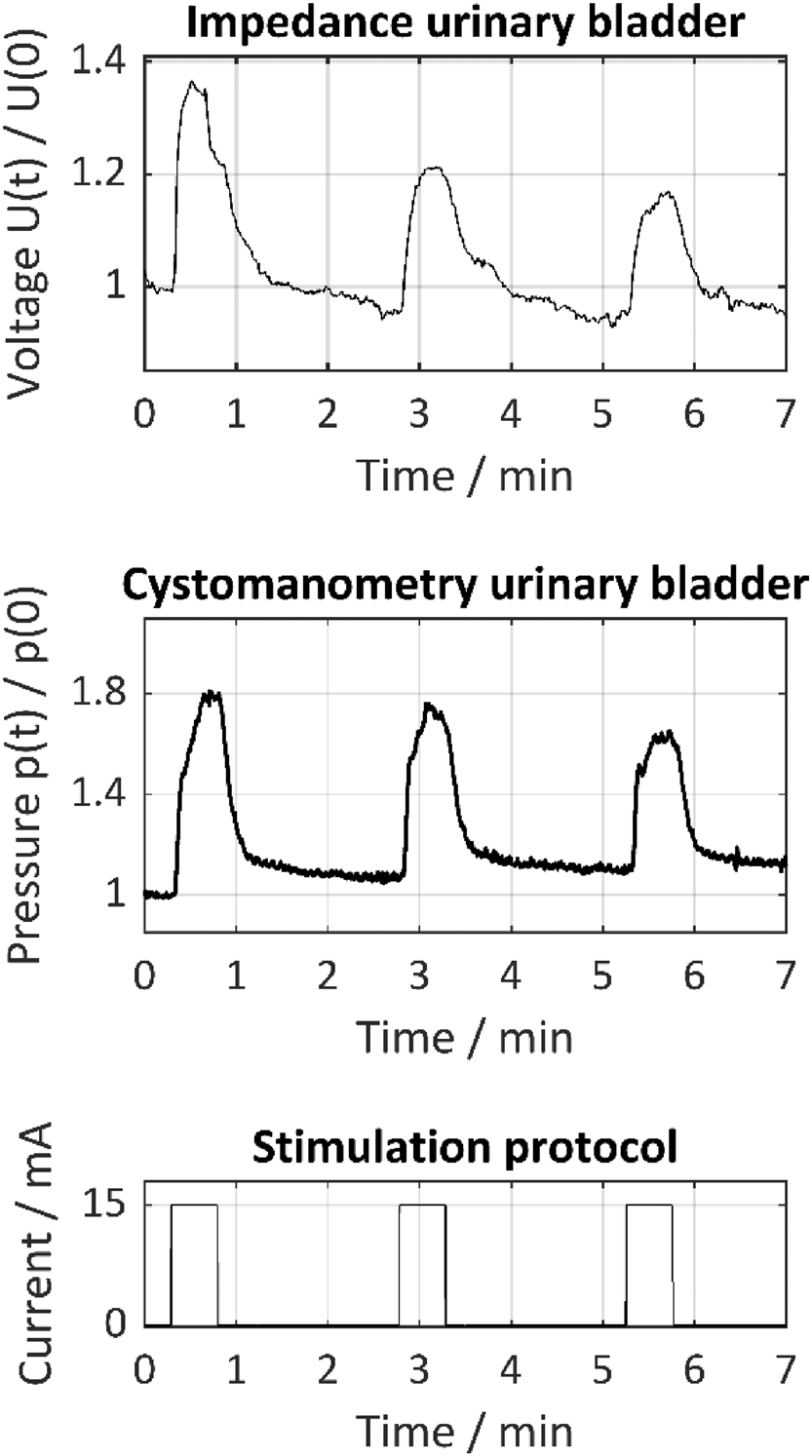
Normalized tissue impedance of the urinary bladder U(t)/U(0) and cystomanometry p(t)/p(0) as a function of time during direct nerve stimulation with 15 mA, 1000 µs pulse width and 30 Hz on the vesicouterine ligament according to the depicted stimulation protocol. Impedance measurement was carried out with four needle electrodes (setup 2). Tissue impedance of the bladder and bladder pressure are consistent and rise during direct nerve stimulation and decrease during relaxation.

Figure 4 shows the results of direct nerve stimulation on the vesicouterine ligament with the Saxophone electrode together with impedance measurement and cystomanometry on the urinary bladder. The normalized change in tissue impedance measured via the voltage U(t)/U(0) and cystomanometry p(t)/p(0) as a function of time during stimulation and relaxation phases is shown. The urinary bladder’s tissue impedance is rising during nerve stimulation of the vesicouterine ligament due to a contraction of the bladder. The end of the stimulation phase correlates with the relaxation of the muscle contraction followed by a decrease in tissue impedance. Performing the test with a filled bladder allowed a comparison with the results of the simultaneously conducted cystomanometric measurements. It turned out that the signal course of the normalized tissue impedance is consistent with that of the cystomanometric measurements and thus in agreement with the literature^15,20^. The main advantage, however, is that impedance measurement does not require bladder filling.

**Figure 5:**
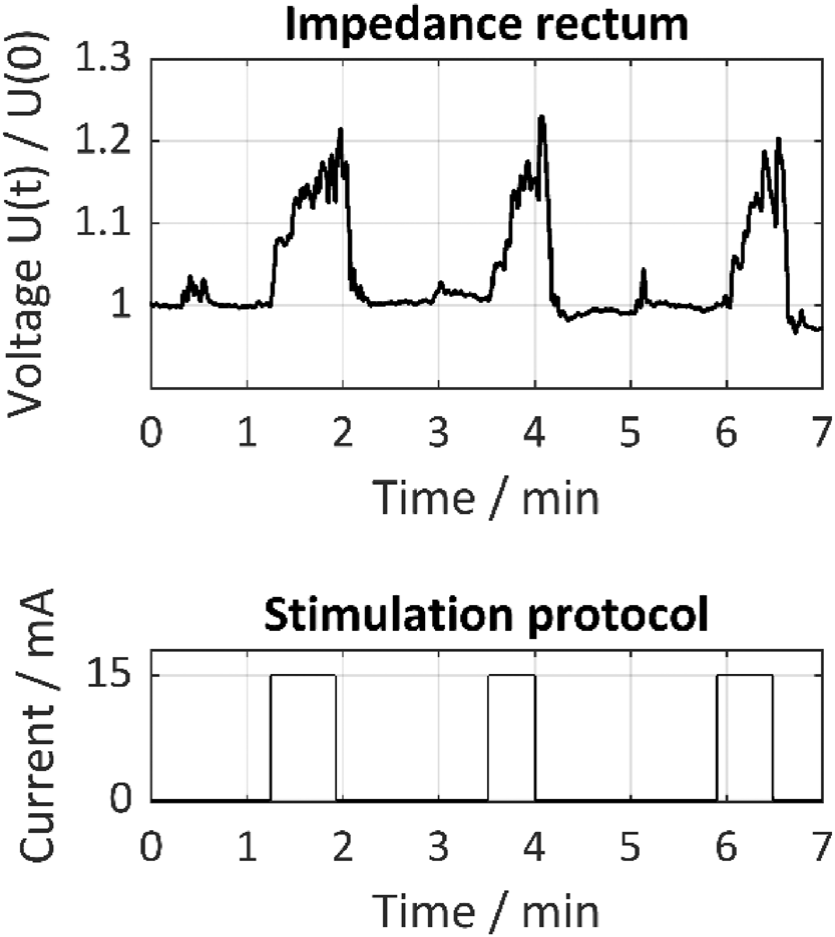
Normalized tissue impedance of the rectum U(t)/U(0) as a function of time during direct nerve stimulation with 15 mA, 1000 µs pulse width and 30 Hz on the paraproctium according to the depicted stimulation protocol. Impedance measurement was carried out with two needle electrodes (setup 1). Tissue impedance rises during direct nerve stimulation and decreases during relaxation and includes superimposed self-activity of the smooth muscle.

Figure 5 shows the results of direct nerve stimulation on the paraproctium with the Saxophone electrode and impedance measurement on the rectum. The normalized change in tissue impedance U(t)/U(0) on the rectum is shown to be equivalent to the urinary bladder. It increases and decreases correlating with the contraction and relaxation of the rectum due to stimulation and relaxation phases on the paraproctium. Compared to the urinary bladder, the rectum shows a self-activity of the smooth muscle, which was also macroscopically apparent and superimposed on the impedance signal course caused by nerve stimulation.

The fixed stimulation positions (vesicouterine ligament for the bladder and paraproctium for the rectum) were used for further evaluation of the method. The influence of the stimulation frequency and the positioning of the recording electrodes on the normalized tissue impedance was analyzed.

**Figure 6:**
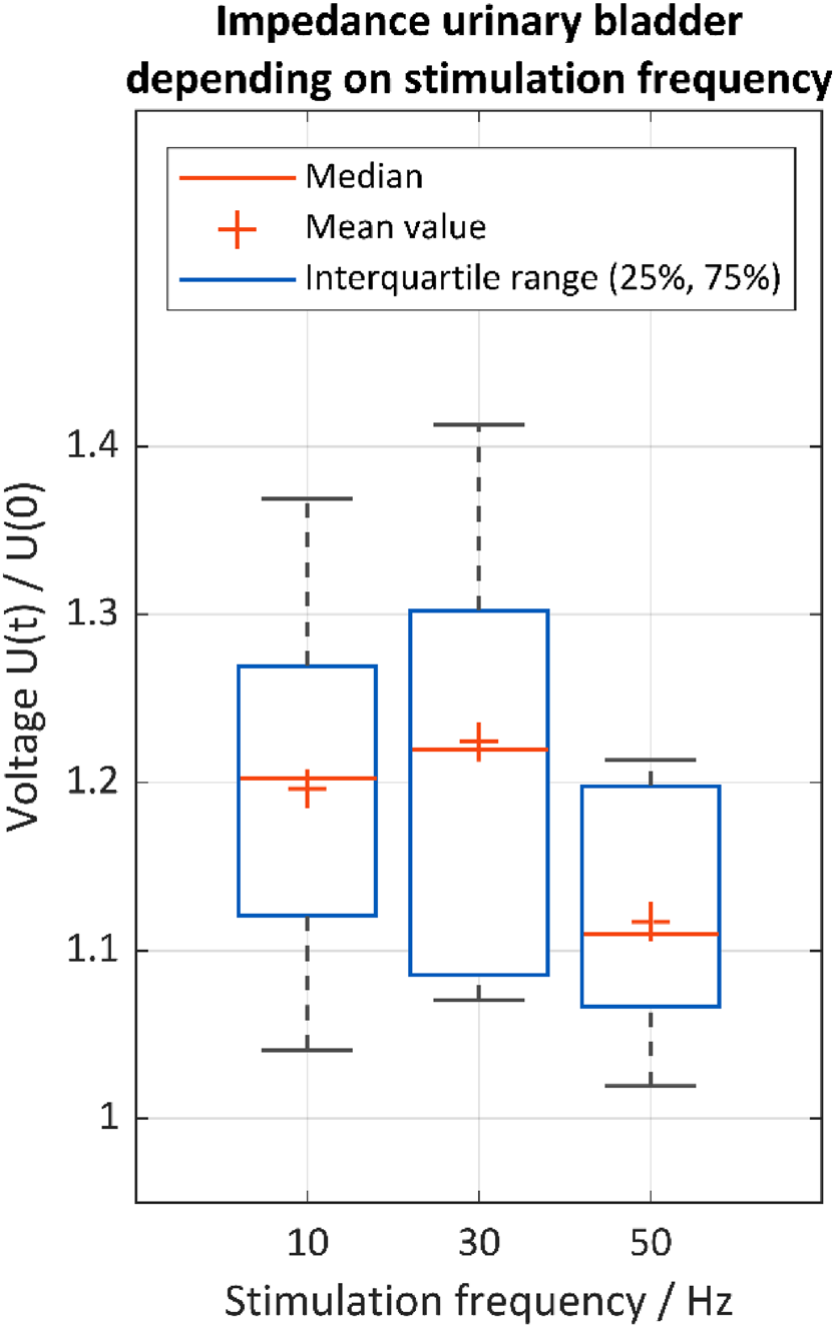
Normalized tissue impedance of the urinary bladder U(t)/U(0) for the stimulation frequencies of 10 Hz (n = 10), 30 Hz (n = 9), 50 Hz (n = 9) (case 1–12). Stimulation parameters: 15 mA, 1000 µs. Stimulation position: vesicouterine ligament, recording electrode setup 2.

Three different stimulation frequencies were included in the stimulation protocol, see Fig. 6. The changes in tissue impedance during the application of 10 Hz, 30 Hz and 50 Hz were compared. A stimulation current of 15 mA, a stimulation pulse width of 1000 µs, a stimulation site at the vesicouterine ligament with the Saxophone electrode and the recording electrode setup with needle electrodes only (Setup No. 2 urinary bladder) were used.

The stimulation frequency of 50 Hz caused the lowest arithmetic mean and median value, which results from the lowest excitation and smooth muscle contraction. The stimulation frequencies of 10 Hz and 30 Hz showed higher dispersions, but also higher arithmetic mean and median values compared to 50 Hz, indicating better excitation and smooth muscle contraction. Since nearly 75% of the values were higher than the arithmetic mean and median value at 50 Hz, both 10 Hz and 30 Hz were considered more appropriate than 50 Hz, despite the higher dispersion. As the stimulation frequency of 30 Hz showed the highest arithmetic mean and median value, it was chosen for further evaluation, which is in agreement with the literature on direct pelvic nerve stimulation in a clinical setting^13,20,23^.

**Figure 7:**
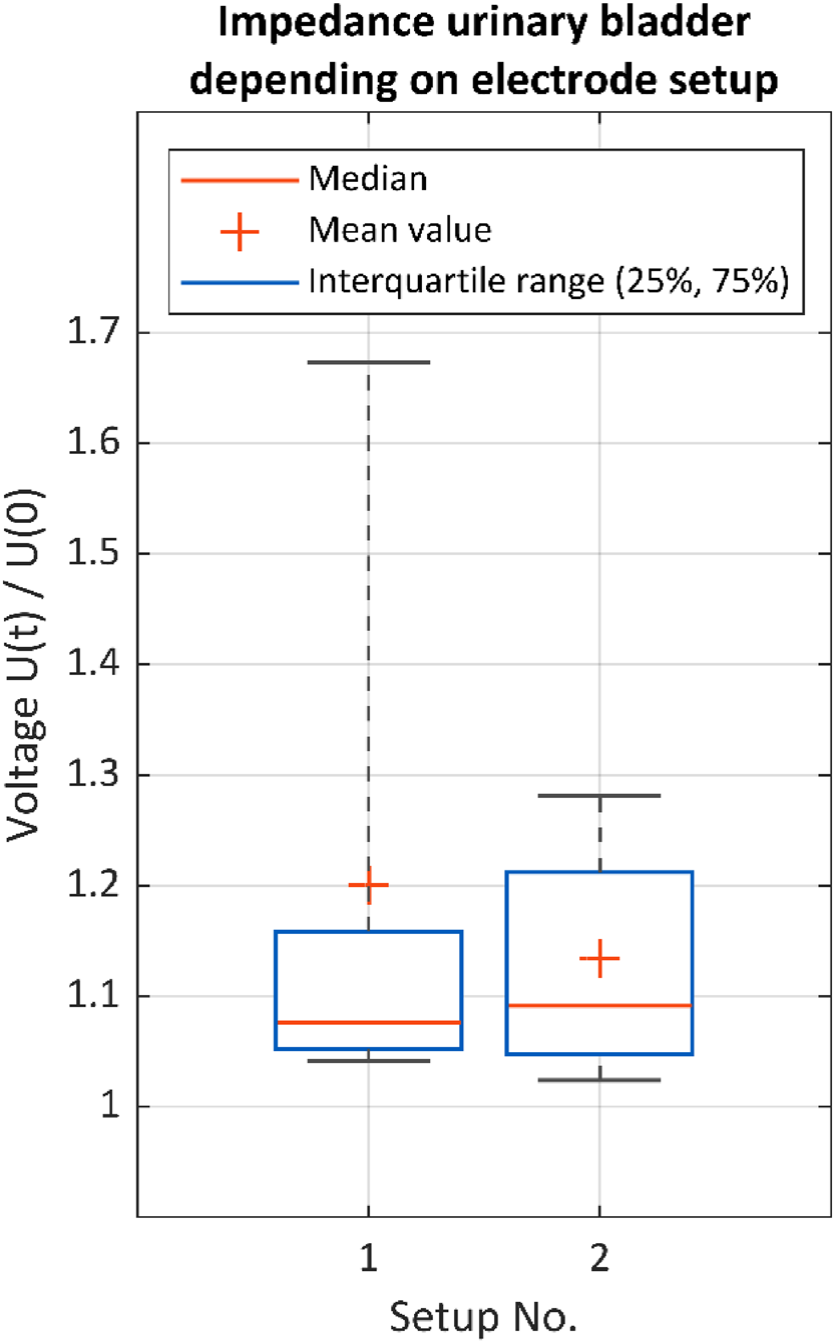
Normalized tissue impedance of the urinary bladder U(t)/U(0) for different electrode setups (case 1–12). Setup 1 (surface catheter electrodes and needle electrodes, n = 29), setup 2 (needle electrodes only, n = 39). Stimulation parameters: 15 mA, 1000 µs, 30 Hz. Stimulation position: vesicouterine ligament with the Saxophone electrode.

As described above, different electrode positions on the urinary bladder and rectum were tested and then evaluated with respect to their usability in a clinical setting. Furthermore, the results of impedance measurement depending on the electrode setups were analyzed. The electrode setups on the urinary bladder were evaluated using the following test conditions: a stimulation frequency of 30 Hz, a stimulation current of 15 mA and the stimulation site at the vesicouterine ligament by the Saxophone electrode. The box plots in Fig. 7 show two measurement cycles for recording electrode setup 1 and setup 2, see also Fig. 3. Pelvic neuromonitoring with electrode setup 1 (catheter electrode/s on the urethra´s internal sphincter muscle and monopolar needle electrode/s on the urinary bladder´s apex) led to a higher mean and an equivalent median value in tissue impedance U(t)/U(0) compared to setup 2 (monopolar needle electrodes on the urinary bladder’s apex and vertex). But the dispersion of the values in setup 1 is much higher than that in setup 2, which is considered negative. Since the positioning of needle electrodes on the urinary bladder´s vertex in humans is not as feasible as the positioning of the urethral catheter electrodes, both setups must be further studied in a clinical setting. However, the results obtained so far suggest that setup 2 is more appropriate because of the much lower value dispersion.

**Figure 8:**
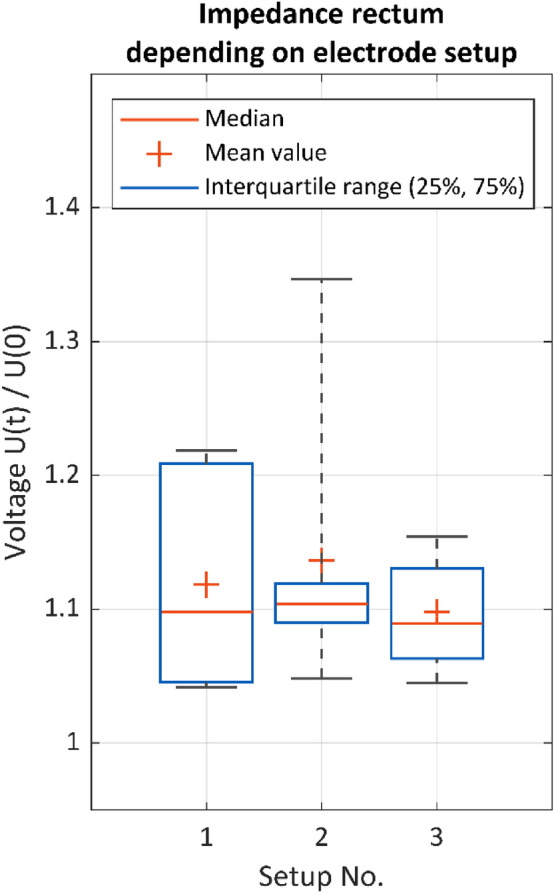
Normalized tissue impedance of the rectum U(t)/U(0) for different electrode setups (case 1–12). Setup 1 (needle electrodes only, n = 12), setup 2 (needle electrodes and surface electrodes, n = 6), setup 3 (surface electrodes only, n = 9), Stimulation parameters: 15 mA, 1000 µs, 30 Hz. Stimulation position: paraproctium with the Saxophone electrode.

To evaluate the electrode setups on the rectum, the following test conditions were used: a stimulation frequency of 30 Hz, a stimulation current of 15 mA and the stimulation site at the paraproctium with the Saxophone electrode. The box plots in Fig. 8 show two measurement cycles for recording electrode setup 1 (needle electrodes only), setup 2 (needle electrodes and surface electrodes) and setup 3 (surface electrodes only) on the rectum. While all three setups show similar results in the arithmetic mean and median value, setup 2 shows the highest arithmetic mean value, but also the highest dispersion of the values, followed by setup 1. Thus, setup 1 and setup 3 are more appropriate than setup 2. But because in a clinical setting a rectal probe is neither desirable nor possible in every case, setup 1 seems to be the most appropriate electrode positioning.

In identifying the superior and inferior hypogastric plexuses no significant differences were found between the results of the measurement cycles with the fixed stimulation positions (paraproctium and vesicouterine ligament) and those during stimulation with the hand probe. A further detailed evaluation or comparison of these values is not meaningful because of the unsteady and varying stimulation positions with the hand probe. However, the hand probe is an essential tool for nerve identification in a clinical setting.

To rule out that the change in tissue impedance is an artefact, a negative control was performed in the wound margin and after deliberately cutting the nerves. In neither case did direct nerve stimulation cause any muscle contraction or a related change in tissue impedance. Also, in none of the four cases in which the influence of muscle relaxation was assessed, did intravenous vecuronium affect any of the results, contraction behavior of the smooth muscle or the feasibility of pelvic neuromonitoring.

### Immunohistochemistry

**Figure 9:**
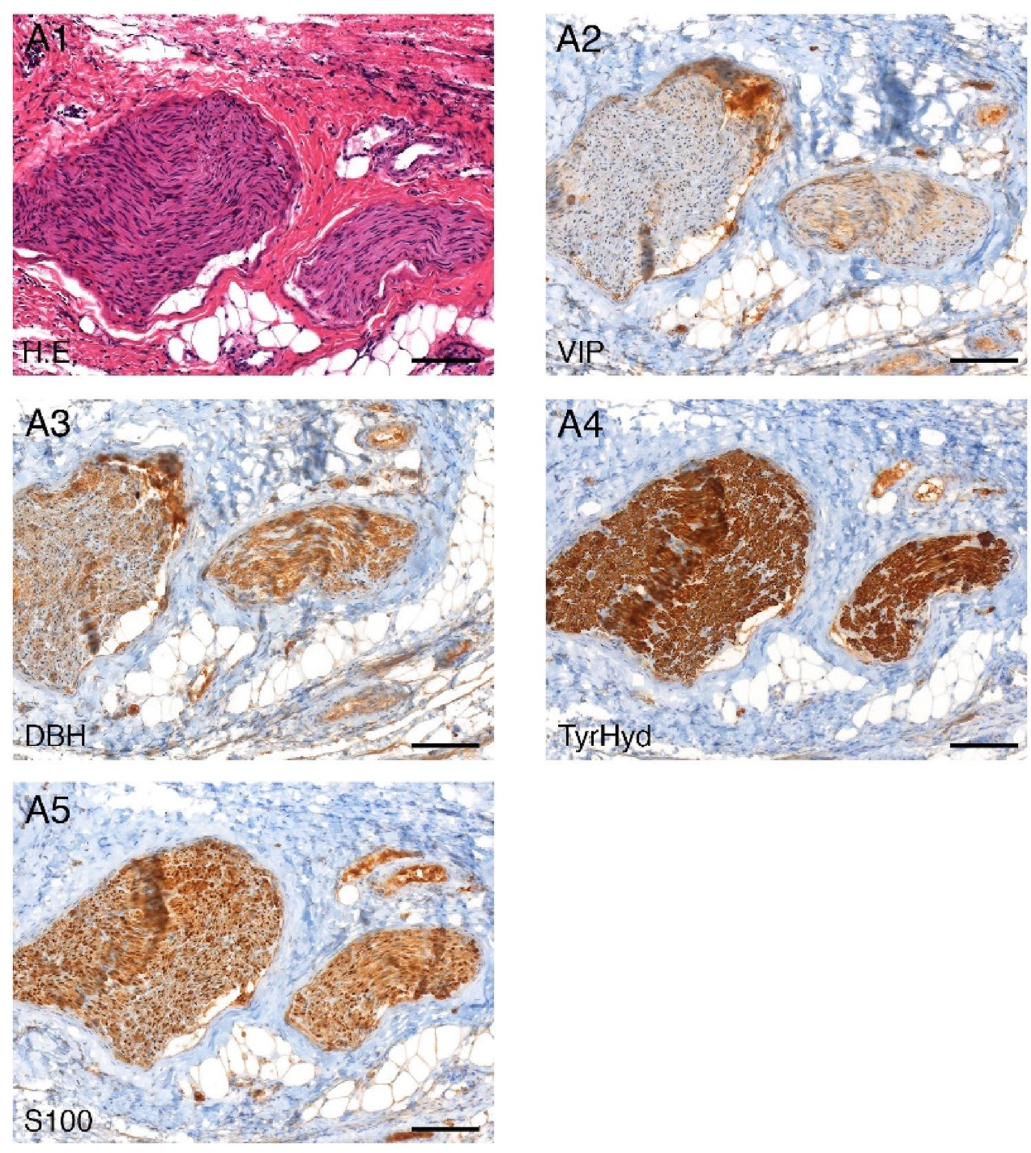
Histology and immunohistochemistry verification of nerve-tissue in the samples taken from the pigs: A1 H.E. staining, A2 vasoactive-intestinal-peptide (VIP) staining, A3 dopamine-ß-hydroxylase (DBH) staining, A4 thyrosin-hydroxylase (TyrHyd) staining, A5 S100 staining, tenfold magnification.

Figure 9 shows the results of the HE and immunohistochemistry of the obtained tissue samples from the pigs which were identified as nerve tissue. The following antibodies were used for nerve tissue identification and differentiation of sympathetic and parasympathetic nerve-fibers: S100-antibody, vasoactive intestinal peptide (VIP) antibody, dopamin-β-hydroxylase (DBH) antibody and tyrosine hydroxylase (TH) antibody. The immunohistochemical staining reaction was benchmarked against appositive controls. From the combination of the markers used it is evident that the samples contained autonomic sympathetic and parasympathetic fibers.

### Macroscopical dissections on human cadavers

**Figure 10:**
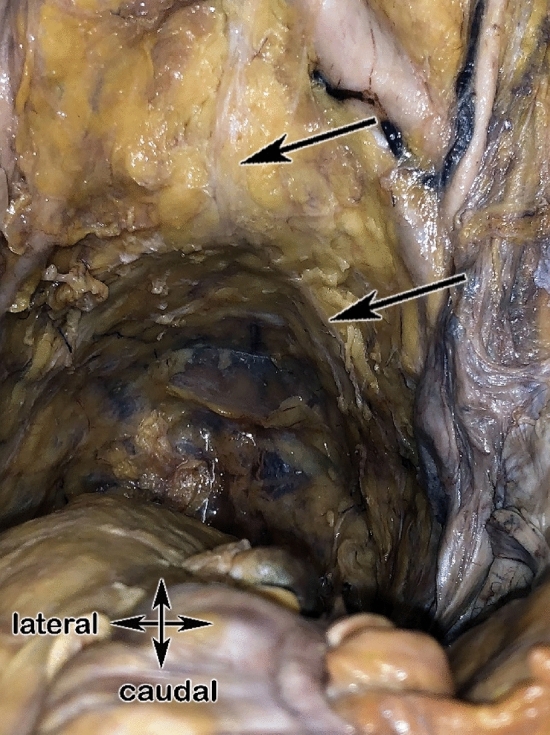
Dissected hemipelvis of a body donor to science showing the electrode placed on the bladder cranially (upper black arrow), inferior hypogastric plexus shown with the lower black arrow.

**Figure 11:**
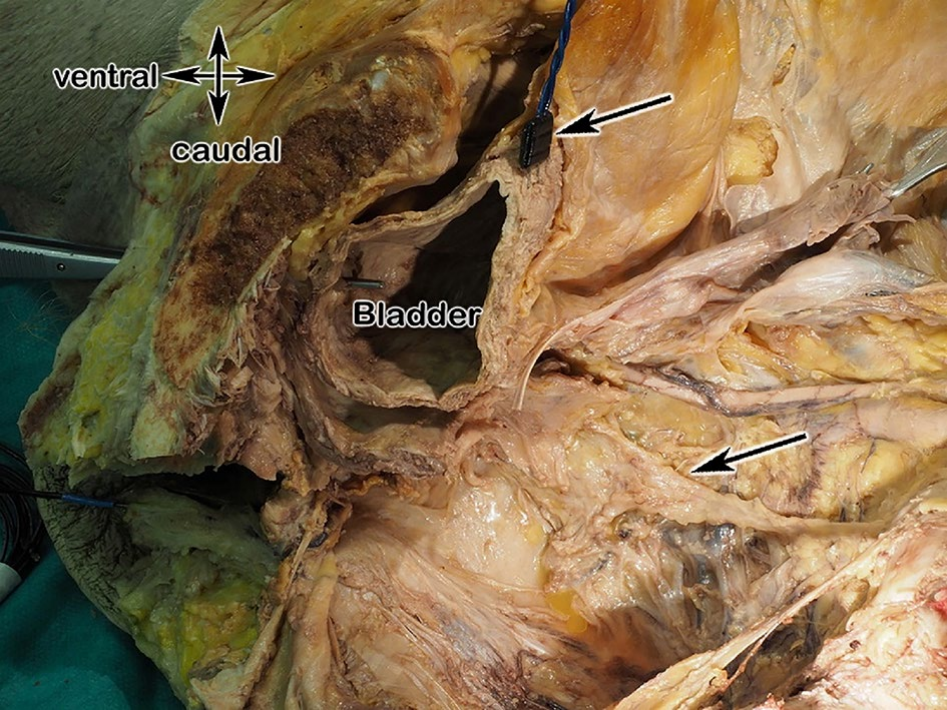
View in the dissected pelvis: superior hypogastric plexus and its bifurcation is shown (black arows).

The dissection was carried out on a female human body donor to science using an anatomical, vertical approach through the abdomen to get into the pelvis. It has been used to visualize the topography of the pelvic autonomic nerves or plexus in humans (Figs. 10 and 11): Superior hypogastric plexus, right and left hypogastric nerves, inferior hypogastric plexus and neurovascular bundles to the organs. Furthermore, the possible topographic position of the electrodes on the urinary bladder for intraoperative pelvic neuromonitoring could be shown.

## Discussion

The study revealed that impedance measurement on the urinary bladder and the rectum is suitable for direct detection of smooth muscle contraction. In all cases in which this method was used autonomic nerve stimulation resulted in a change in tissue impedance. The investigation also confirmed the technical feasibility for all analyzed electrode setups and stimulation sites. These findings suggest that the interpretation of changes in tissue impedance of the smooth muscle in combination with direct pelvic nerve stimulation could be a new method for intraoperative pelvic neuromonitoring, allowing to identify nerves at risk during low anterior resections, especially LAR with TME for rectal cancer.

Current research on bioimpedance measurement mostly addresses quantitative methods like bioimpedance spectroscopy to determine or characterize a specific tissue type or cell status^24,25^. Detection of smooth muscle contraction by means of quantitative determination of an absolute impedance value of the smooth muscle requires the same electrode-tissue contacts, identical electrode types and equal measurement electrode distances in every individual and surgical procedure. A quantitative evaluation would also require a precise correlation between impedance values and a certain status of muscle contraction valid for every individual. However, this is not feasible in a clinical setting due to the above-mentioned degrees of freedom, especially the varying positioning of measurement electrodes caused by anatomical differences in individuals. Thus, a qualitative evaluation of the tissue impedance in comparison to the status before contraction was chosen for our method.

In general, the impedance of biological tissue depends on the electrolytic characteristics of the fluid spaces between the cell membranes and the capacitive characteristics of the cell membranes themselves^22^. Assuming that the alternating structure of dense bodies and connective tissue generates capacitive properties in the smooth muscle cell, water and proteins generate resistive properties. Due to geometric changes during muscle contraction, the distances in the alternating structure of dense bodies and connective tissue are shortened, water can be displaced by changing fluid spaces, therefore the capacitive and resistive properties of the muscle change, resulting in a change in tissue impedance.

Due to the successful negative control, false positive stimulation events can be excluded, which confirms the accuracy of the method. This was the most important goal of this study.

Further evaluation of the method adopted in this study showed that the stimulation frequency of 30 Hz is the most appropriate. Kauff and Kneist et al., who have published research on pelvic neuromonitoring since 2011, have also reported the usage of the stimulation frequency of 30 Hz in several studies. Stimulation currents in the range of 1–25 mA were described^19^, whereby a current of 6 mA was applied in most studies^20,21,26^, which also corresponds to the approach in this study.

However, signal analysis within this study had some limitations. It was found that the number of excitable nerve branches reached by the permanent stimulation electrode in a fixed position is only steady when tests are repeated in the same individual without changing anything. But when the same setup is used in another individual, the number of reached excitable nerve branches varies even if the same anatomical landmark is stimulated. Uncertainties are the tissues surrounding the nerve that cannot and should not be separated from nerve tissue, leading to test conditions which are not completely constant. Additional factors influencing the stability of test conditions are varying physiological and anatomical conditions, resulting in varying muscle contraction behaviors. Thus, it is not possible to create identical test setups with the same positions of stimulation and recording electrodes and similar muscle reaction. The signal analysis with data collected from all animal cases gives us information about the feasibility of the method but reveals uncertainties mainly in the comparison of impedance values. Therefore, further signal interpretation and evaluation is mandatory during transfer to a clinical setting.

In applying this method, two main problems were observed. One of the problems is the precise positioning of the electrodes, which is essential for obtaining reliable results. When the contractile muscle section is not covered by the two measurement electrodes, the change in tissue impedance is not detectable although the muscles did contract. Therefore, a slightly diagonal positioning from the urinary bladder’s apex to the vertex is helpful, because then nearly the whole contractile muscle of the urinary bladder is covered. On the rectum, the electrodes should be positioned on the contralateral side to the electrodes in the lower rectum so that the rectum, the sigmoid and the anal sphincter muscles are covered.

The other problem is the surgical manipulation associated with the procedure of LAR with TME itself. Our tests showed that mechanical manipulations cause artefacts in the impedance signal which can complicate signal interpretation. But because the signal characteristics of a physiologically caused change in tissue impedance are different from those caused by surgical manipulations, signal interpretation becomes possible.

Pelvic neuromonitoring has been subject of scientific research for about three decades. In 1989 Horgan et al. published their work on pre-, post- and intraoperative anal manometry and presacral nerve stimulation in cases of low ante^27^rior resections^28^. Since then, several approaches have been evaluated and mainly one method has been adopted until today: intraoperative cystomanometry and online-processed electromyography of the internal anal sphincter (IAS) during autonomic nerve stimulation with a hand-guided probe. A series of studies has been published fby the research group Kauff and Kneist et al.^15,20,23^. Recently published studies indicate that pelvic intraoperative neuromonitoring performed with cystomanometry and EMG of the IAS is associated with lower rates of new-onset urinary dysfunction and fecal incontinence^21,26^. The practical application of this method requires filling the urinary bladder with Ringer’s solution for cystomanometry and precise positioning of the electrodes on the internal anal sphincter, which has to be performed under endosonography in case needle electrodes are used^29^.

In contrast, our study showed that tissue impedance measurement is suitable for detecting contraction of the urinary bladder without the need of time-consuming bladder filling. Moreover, detecting activity of the anal sphincters does no longer require inserting the needle electrode precisely into the sphincter muscle. It is sufficient to have the internal or external anal sphincter within the impedance measurement region without the need of endosonography. Additionally, tissue impedance displayed as a function of time has turned out to be a direct and easily interpretable indicator of the activity of the urinary bladder and the rectum.

Other methods for pelvic floor neuromonitoring are bulbocavernosus reflex (BCR) measurements, recording of transcranial motor evoked potentials (tcMEPs) and somatosensory evoked potential recordings (SSEPs) from the pudendal nerve^30^. The bulbocavernosus reflex (BCR) is a method for functional control of the reflex arc at the sacral spinal level S2-S4 via afferent and efferent fibers of the pudendal nerve from the clitoris/glans penis to the motor pelvic floor muscles. This method is typically used for spinal procedures at the sacral level and for procedures in the lesser pelvis^16,30^. BCR is not used to monitor pelvic autonomic nerves such as the superior or inferior hypogastric plexus, which is the most important difference from the impedance measurement method presented. TcMEPs used for pelvic neuromonitoring include transcranial stimulation of the motor cortex and MEP measurement at the external urethral sphincter (EUS) and external anal sphincter (EAS), both of which are motor muscles innervated by the pudendal nerve. This method is useful for sacral spine procedures as well as pelvic procedures where the pudendal nerve is at risk^16,30^. Direct identification of the pelvic autonomic nerves in the surgical area is not possible with the MEP method. Pudendal SSEPs are elicited by anal, penile, or clitoral stimulation and recorded by averaging 200–300 synchronous EEG sweeps—random EEG activity is reduced, and the evoked potential is filtered. Consecutive and reproducible EPs are interpreted using amplitude and latency analysis^18,30^. This method is used for functional control of sensory fibers of the pudendal nerve; identification of autonomic nerves in the surgical area is not possible with this method.

In this preclinical study the feasibility of a new method for pelvic intraoperative neuromonitoring was analyzed. The results indicate a reliable identification of pelvic autonomic nerves during low anterior resections (LAR), thus pelvic nerve damage might be prevented in the future. The transfer of our results to a clinical setting on humans is in progress within a clinical trial (German Clinical Trials Register DRK S00017437).

## Data Availability

All data generated or analysed during this study are included in this published article (and its Supplementary Information files).
